# Modeling Capacitive Low-Power Voltage Transformer Behavior over Temperature and Frequency

**DOI:** 10.3390/s21051719

**Published:** 2021-03-02

**Authors:** Alessandro Mingotti, Federica Costa, Gaetano Pasini, Lorenzo Peretto, Roberto Tinarelli

**Affiliations:** Department of Electrical, Electronic and Information Engineering, Guglielmo Marconi Alma Mater Studiorum, University of Bologna, Viale del Risorgimento 2, 40136 Bologna, Italy; federica.costa13@unibo.it (F.C.); gaetano.pasini@unibo.it (G.P.); lorenzo.peretto@unibo.it (L.P.); roberto.tinarelli3@unibo.it (R.T.)

**Keywords:** capacitive divider, modeling, setup, procedure, voltage divider, capacitor, supraharmonics, temperature, frequency, partial discharges

## Abstract

The use of capacitive dividers (CDs) in medium-voltage (MV) networks started as simple voltage detectors and as rough voltage measurement instruments for protective purposes. Now, with the spread of intelligent electronic devices and renewable energy sources at the distribution level, capacitive dividers are designed and installed to perform accurate voltage measurements. Such a requirement is mandatory when the power quality has to be assessed. Therefore, CDs are currently being used either for power frequency or for high-frequency (supraharmonic- or partial-discharge-level) measurements. In this paper, typical off-the-shelf CDs are studied and modeled to understand how they behave in a wide range of frequencies and when the temperature varies. To this purpose, specific setups and tests have been developed and performed. From the results, it is clear that with proper modeling of CDs, it is possible to exploit them for measuring phenomena in a wide range of frequencies, including the effects due to temperature variations and self-resonances.

## 1. Introduction

The measurement of electrical quantities, voltages, and currents requires accurate and reliable devices. This role has been played by instrument transformers (ITs) since the first electric grids were developed at the end of the 19th century. Voltage and current transformers (VTs and CTs, respectively) scale the primary voltage or current, respectively, to a value suitable for being acquired by the acquisition system or for being sent to the next element of the measurement chain.

Since their first development, power grids underwent a huge improvement, and this will not stop even in the near future. In the same way, ITs experienced incredible progress to become capable of adapting their measurement capabilities to a grid that is constantly evolving.

From the standardization perspective, this can be noted from the movement of the old IEC 60044 series to the new set of documents included in the IEC 61869 series [1,2]. Those two IEC series aim to standardize all the aspects related to ITs, from research to the final users’ perspectives, as is better described in what follows.

Among ITs, until 10–20 years ago, the most installed and efficient type was the inductive one, always preferred by system operators (SOs) for its reliability and ease of construction compared to other solutions. As a result, researchers did and are currently doing excellent work in studying the main properties and capabilities of ITs. For example, for the sake of completeness, VT modeling has been tackled in [3,4] while CTs have been modeled in [5,6]. Finally, in [7,8,9], a new IT modeling approach, a mathematical-based one, and a design-based model have been presented, respectively.

Another important aspect of ITs to be studied is their accuracy and how it can be improved. In particular, methods and procedures to evaluate IT accuracy (in both normal and distorted conditions of the network) have been developed in [10,11,12]; the role of the software in the accuracy evaluation process has been studied in [13]. In addition, and of high relevance for SOs, in [14,15,16], three different ways of how low-accurate measurements may affect the correct operation of the network and of the services implemented to manage it have been described.

As is well known, IT accuracy is not just influenced by the transformer design. In fact, their behavior may change due to external phenomena and operating conditions different from the rated ones. For example, low levels of power quality (PQ), temperature, and electric fields are among the most common causes of IT malfunctioning and accuracy degradation. The effects of such influencing factors are studied in [17,18,19,20], [21,22,23], and [24,25,26], respectively.

In parallel to scientific research, IEC standards are collecting, supported and guided by the technical committees (TCs), all the literature and experience to produce new documents that deal with all possible situations that may occur when an IT is operating. In this specific case, IEC 61869, as anticipated before, covers inductive CTs and VTs in the document-2 and document-3 [27,28], respectively, while the emergent generation of electronic and low-power instrument transformers (EITs and LPITs) is contained in document-6 [29] (general purpose) and in document-7 to document-13 for each specific type of transformer.

Among such promising types of LPITs, there is the subject of this work. In particular, the passive capacitive low-power voltage transformer (LPVT) is studied and modeled. This peculiar type of LPVT changed the way to perceive voltage measurements; therefore, Section 2 is dedicated to its features and description. However, from a wider perspective, the introduction of LPITs among measurement instruments was a breakthrough innovation for SOs (for both distribution and transmission networks). At a glance, it can be stated that LPITs, compared to typical inductive ITs, feature smaller dimensions, lower weight, low cost, ease of installation, etc. Therefore, LPITs brought a completely new set of capabilities, to be used and to be studied, that changed the way of measuring electrical quantities. Of course, even LPITs have their drawbacks, and they are not immune to all external influence factors. However, this new type of transformers opened a new set of scenarios and potential applications that were not possible before.

One of these scenarios has given the motivation for this work to examine in depth some aspects related to LPVTs. In detail, the idea is to extend the operating conditions of the LPVT capacitive dividers, which are designed to work from the nominal frequency (50/60 Hz) up to the PQ frequency range (50th harmonic). The aim is to exploit the in-field large availability of installed capacitive dividers to perform measurements outside their PQ frequency range, hence up to the supraharmonics range (<150 kHz) or even up to some megahertz, to investigate their potential for measuring partial discharges (PDs). Of course, the frequency behavior limited to some portion of the bandwidth is something that has been already treated in the literature (see a deeper analysis in Section 2). The added value of this work consists of focusing on the behavior of the capacitive divider from 50 Hz to 1 MHz, when the temperature varies from −5 °C to 40 °C, highlighting the design aspects that may extend the capacitive divider’s (CD) capabilities, allowing SOs to have a single device capable of collecting more information from the performed measurements.

Therefore, this work assesses the behavior vs. frequency and vs. temperature of two medium-voltage (MV) off-the-shelf capacitive dividers that are typically installed in the Italian secondary substations. The obtained results allow modeling the CDs’ behavior, highlighting the pros and cons of using the CDs in the in-field environment and at different frequency ranges.

The remainder of the work is structured as follows: Section 2 deals with capacitive dividers, detailing their features and operation in the main relevant applications. The modeling, and hence the core of the work, of capacitive dividers is treated in Section 3. In particular, the tests and the associated measurement setups are described and discussed. Test results are presented in Section 4, including the comments emerging from them. Finally, a brief summary of the achievements and a conclusion of the work are given in Section 5.

## 2. Low-Power Voltage Transformers: Capacitive Dividers

### 2.1. Working Principle

**Figure 1:**
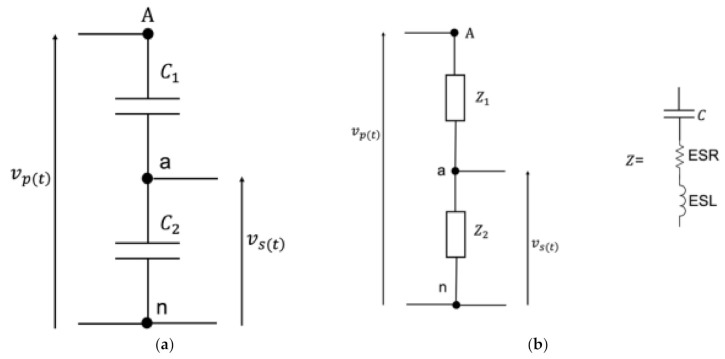
(**a**) Ideal representation of a capacitive divider. (**b**) Real representation of a capacitive divider, which is made of impedances.

Whether parallel or cylindrical plate based, capacitive dividers operate according to a very simple working principle. In fact, as depicted in Figure 1a, two capacitors $C_{1}$ and $C_{2}$ are sufficient to scale a voltage proportional to the capacitors’ values. More specifically, in a set of two capacitors, the smaller one is subject to the higher voltage drop. Therefore, according to Figure 1, $C_{1}$ is the smaller capacitor of the circuit, allowing one to reduce the primary voltage $v_{p}\left(t\right)$ to the secondary voltage $v_{s}\left(t\right)$ as:

(1) $$v_{s}\left(t\right)=v_{p}\left(t\right)\frac{C_{1}}{C_{1}+C_{2}},$$

The reason for this apparently controversial behavior, compared to a divider consisting of resistors, is described by the relation between the capacitance and the reactance $X_{C}$:

(2) $$X_{C}=\frac{1}{\omega C},$$

which better represents the ohmic quantity capable of reducing the voltage. From Equation (2), it can be also noted that the reducing strength of a capacitor depends not only on its capacitance value but also on the rated frequency (ω=2πf) of the considered application.

A brief note should be included here to analyze the features of a CD compared to other technologies, like the resistive one. In particular, resistive dividers (RDs) are not affected by the operating frequency except for the white noise introduced by the resistance value (which increases with the resistance). Furthermore, RDs are linear like CDs; however, they allow measuring DC components, which is not possible with a capacitive technology due to its operating principle. On the contrary, some drawbacks of resistive technology are (i) the heating dissipation, which is an intrinsic property, due to the current flowing through the divider, and (ii) the influence of temperature, which causes changes in the resistance values of the RDs. Therefore, the use of RDs and CDs is almost comparable, but it strictly depends on the final application.

What is presented up to now holds for an ideal representation of a capacitive divider. However, its description is typically improved when including the non-idealities of the capacitor. The enhanced model is presented in Figure 1b, in which it is clear that the capacitor is now described by an impedance ($Z_{1}$ and $Z_{2}$ for the primary and secondary sides, respectively) consisting of two additional components: an equivalent series resistor (ESR) and an equivalent series inductance (ESL). The former component includes in the model the heat dissipated by the divider due to the presence of metallic parts and electrodes in the capacitor. As for the ESL, instead, it considers the parasitic inductance that, depending on the application frequency, may lead to the auto-resonance phenomenon of the capacitor, resulting in a drastic change in the CD operation.

Note that for the purpose of this work, the two equivalent circuits presented in Figure 1 represent a sufficient level of detail. However, more complex and accurate descriptions of a capacitor are well described and used in the literature (e.g., like those presented in [30,31,32]).

Summarizing and concluding, the choice of a CD for a specific application is reduced to the selection of the CD ratio, which is described by its ideal $k_{id}$ or real $k_{re}$ formulation as:

(3) $$k_{id}=\frac{C_{1}+C_{2}}{C_{1}},\;k_{re}=\frac{Z_{1}+Z_{2}}{Z_{2}}.$$

### 2.2. Modeling

**Figure 2:**
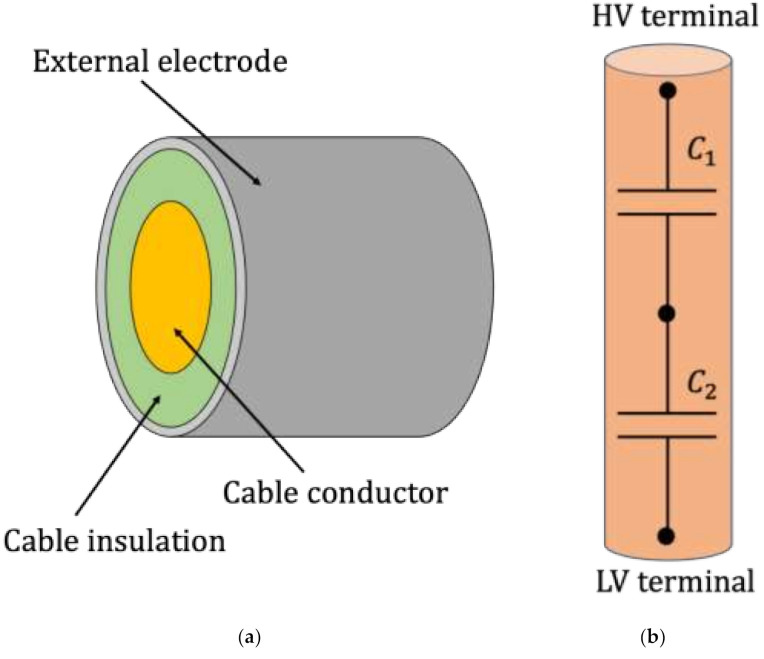
(**a**) Simple representation of a cylindrical capacitive divider. (**b**) Simple representation of a common capacitive divider.

To analyze how capacitive voltage dividers are modeled, it is first necessary to distinguish between the two main typologies that can be found in the literature and in the market. From Figure 2, it is possible to appreciate the cylindrical ring CD on the left, while the most common solution with parallel plates and aligned capacitors is depicted on the right side. The cylindrical CD is commonly installed on cables, which constitute the inner electrode of the primary capacitance of the divider. Then, the cable insulation constitutes the dielectric of the divider, and finally, an external electrode layer completes the primary capacitor. Of course, the divider is complete with a secondary capacitor, which has been omitted for the sake of simplicity.

As for the CD on the right side of Figure 2, a case containing the CD is completed with a high-voltage (HV) terminal on the upper part and with a low-voltage (LV) terminal on the bottom part of the divider. This solution is commonly installed at the cable terminations or on the power transformers. It is worth being reminded that in the past, the main purpose of voltage sensors in MV networks was of indicating the presence of a voltage and controlling protective devices. Therefore, their new measurement role, of helping SOs in the PQ evaluation process, results in a lack of the literature on modeling CDs. In addition, more references can be found on cylindrical-shaped CDs than for the second type (which is the aim of this work).

From the literature, in [33], the capacitive voltage divider technique has been used to design a capacitive sensor. However, such a technique is based on active circuitry that modifies the potentials among the nodes of the dividers; hence, it is not applicable to passive devices. Furthermore, the presented technique is incapable of removing the effect of stray capacitances from the capacitor computation. In [34], a passive-damper filter was developed to stabilize a capacitive-coupling substation. The aim was to reduce the disturbances associated with the reduction in voltage from the high to the medium level. However, the solution is significant in terms of dimensions, cost, and impact on the grid; therefore, unfortunately, it is not suitable for a typical secondary substation, which is affected by limited space for installing new devices and by low investment per substation availability.

Several multi-objective algorithms were used in [35] to model and optimize a capacitive voltage divider that will be used for switchgears. The research was completed with some test simulations and required high computational effort. To overcome the limitations of a pure capacitive voltage divider, in [36], a wide-band CD was modeled adding resistors in parallel to both the primary and the secondary capacitor. Finally, they evaluated the most efficient shape for a CD that does not suffer from parasitic effects.

As for the CD mounted on MV cables, in [37], a model was developed to describe the CD behavior considering the temperature variations in the XLPE insulation of the cable. In [38], instead, a CD for high-pulse HV applications was developed and prototyped. The divider was modeled and designed specifically for the detection of high pulses and protective purposes. 

Note that from what is presented above, it is not easy to find studies that treat the modeling of CDs in all their aspects. No models for MV capacitive dividers have been found treating CD behavior when the operating conditions vary. Consequently, this aspect is tackled and described in Section 3.

### 2.3. Influence Factors

In addition to a model that is capable of reproducing the behavior of a capacitive divider operating at its rated conditions, it is necessary to understand how such a behavior is affected by any influencing factor. As a matter of fact, each electric asset, including CDs, is affected by specific quantities (like temperature, humidity, electric field, geometry, etc.) depending on its working principle and design technology. For example, cable joints suffer from temperature variations and from environmental conditions [39,40]. Power transformers, instead, are affected by vibrations, PDs, and electrical stresses [41,42,43]. Finally, voltage insulators are really sensitive to specific environmental conditions, like humidity, pollution, temperature, etc. [44,45,46].

Turning to CDs, many factors influence their correct operation. Frequency, for example, is one quantity that has to be considered when selecting the appropriate CD. Therefore, a specific paragraph has been written in Section 2.4. Among the other quantities, geometry, eccentricity, and cable dimensions are the most studied for the cylindrical ring CDs mounted on cables. As presented in [47,48], such quantities may significantly alter the behavior of the CD, resulting in large discrepancies between the rated and computed measurement accuracies.

Pollution and moisture are other critical quantities that modify the accuracy of CDs; in fact, in [49], a solution is presented based on the introduction of guarding electrodes to minimize the disturbances introduced by the two quantities.

Another undesired situation during the operation of a CD is the presence of stray capacitances. As is well known, points or nodes subjected to different voltages generate, between them, parasitic capacitances that cause leakage currents that modify the equivalent circuit of the entire CD. Consequently, in [50,51], a new way to quantify and model the stray capacitances to remove their contribution from the overall accuracy evaluation of the CD has been described.

Finally, temperature can be considered the most (or at least among the top) influencing quantity that affect a CD. In particular, temperature affects all kinds of LPVTs in a more significant way compared to the legacy inductive ITs. Therefore, the temperature effect on LPITs, and CDs, was tackled by the literature [47]. The main effect of temperature, either low or high (compared to the rated 20–25 °C) is to move the accuracy of the divider outside its accuracy class (AC) limits. Therefore, manufacturers and researchers must work on the design of CDs to avoid such a phenomenon.

In this work, temperature is one of the two influence quantities that have been considered to understand the behavior, and in particular the transformation ratio, of two off-the-shelf capacitive dividers.

### 2.4. Operation vs. Frequency

Frequency is the second influencing quantity that is considered in this work, with temperature, for CD modeling. In particular, a wide range of frequencies is considered, from 50 Hz to 1 MHz, to understand the capabilities of two off-the-shelf dividers at frequencies different from those for which they have been designed.

Two comments are needed before moving on with the discussion. First, from the ideal representation of the CD presented in Figure 1, it might seem strange to deepen the discussion on the behavior vs. frequency of the CD (like it is crucial for other ITs [52,53,54,55]). In fact, from Figure 1 and from the left side of Equation (3), it is clear that the divider behavior is not affected by frequency changes. However, the ideal behavior cannot be considered valid for typical available CDs.

Second, studies on very large frequency ranges, as described below, are not very common. This is simply due to the fact that studies focus on the frequency range of interest; hence a complete overview is not always necessary.

Therefore, in [56], the frequency behavior of CDs in the PQ frequency range was studied. The work focused on harmonic component detection and evaluation. A higher frequency range, 10 kHz to 10 MHz, was studied in [57], where a dedicated measurement setup and experimental procedures were defined and proposed to test CDs at those frequencies. Finally, the design of new CDs that can operate from 2 MHz to 3 GHz was treated in [58]. The aim was to find design and technological solutions suitable for measuring ultra-fast voltages (μs and ns phenomena). A similar study but on cylindrical CDs mounted over cables was performed in [38].

## 3. Test Description for CD Modeling

This section describes the test designed and performed to assess CD behavior and to obtain a sort of modeling vs. temperature and vs. frequency. In particular, Section 3.1 describes the divider ratio measurements vs. temperature and vs. frequency, whereas Section 3.2 presents a further test where the CD’s impedance (with a focus on the capacitance) has been evaluated to better understand the CD’s behavior.

### 3.1. Measurements of the Transformation Ratio vs. Temperature and vs. Frequency

The main test performed in this work is described in this section. The aim was to measure the transformation ratio of the two off-the-shelf dividers at various frequencies and at different temperatures (the two quantities are simultaneously applied). Therefore, Section 3.1.1 is dedicated to the measurement setup description, and Section 3.1.2 describes how the tests have been performed.

#### 3.1.1. Setup

**Figure 3:**
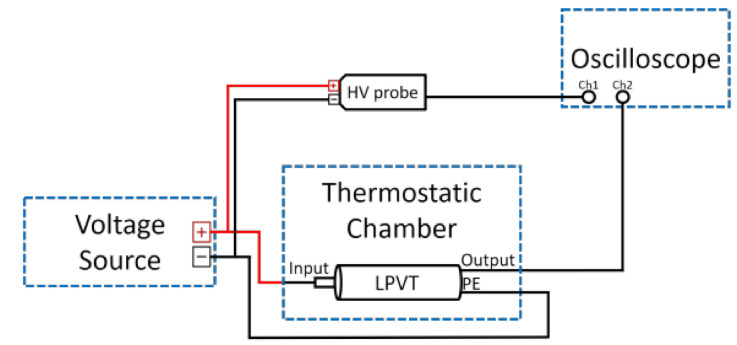
Transformation ratio measurement setup used for testing vs. temperature and vs. frequency.

The measurement setup developed to perform tests over temperature and frequency is illustrated in Figure 3.

It consists of:

- A Datron Wavetek 4800 multifunction calibrator used as a voltage source. The calibrator is used to supply the LPVTs at a maximum voltage of 200 V for frequencies ranging between 10 Hz and 100 kHz. However, due to limitations of the calibrator itself, a maximum voltage of 20 V can be supplied for frequencies above 100 kHz. For this reason, Table 1 and Table 2 provide the accuracy for the two voltage ranges. The frequency uncertainty is less than 100 ppm for all ranges.
- A thermostatic chamber. It allows to set temperatures in the range from −40 °C to +180 °C.
- A high-voltage differential probe. It features four attenuation ranges at ×100, ×200, ×500, and ×1000, corresponding, respectively, to a maximum rms (root mean square) voltage input of 230 V, 460 V, 1140 V, and 2300 V. The accuracy is ±2% for all attenuation ranges.
- Oscilloscope Tektronix MSO58. It has a bandwidth of 350 MHz, and the ADC (Analog to digital converter) resolution is 12 bits. The input impedance consists of 1 MΩ and 13 pF.
- Two passive off-the-shelf capacitive LPVTs under test whose characteristics are listed in Table 3. Precisely, the following specifications are reported: primary and secondary rated voltages ($V_{pr}\left(t\right)$ and $V_{sr}\left(t\right)$, respectively), accuracy class (AC), rated operating frequency ($f_{r}$), and an operating temperature range in °C ($T_{R}$). Considering that the aim of this work is not to evaluate the performance of a specific product, the two LPVTs are referred to as A and B from here on.

### 3.1.2. Test Description

To perform transformation ratio tests, the setup depicted in Figure 3 was exploited as follows. 

The voltage source provides an AC voltage of 200 V for frequencies ranging between 10 Hz and 100 kHz. For higher frequencies, a maximum voltage of 20 V was applied (due to the calibrator limitations). However, it should be highlighted that the harmonic components of a 50 Hz signal are always lower than the rated voltage by a few percentage points. Therefore, the adopted voltage test levels are aligned with practical situations. As for the 50 Hz component, considering the capacitive nature of the devices, no non-linearity effect is expected. For the sake of clarity, a preliminary 50 Hz test of the device under a test rated voltage was performed to guarantee that the divider ratio is not affected by the voltage amplitude.

To measure these voltages, the differential probe was set to an attenuation factor of ×100, standing a peak-to-peak voltage input of 700 V, which corresponds to a maximum rms input of 230 V.

To acquire the input $\left(V_{p}\right)$ and the output voltage $\left(V_{s}\right)$ of the LPVT after being scaled, the oscilloscope was used. Low-pass filters at 5% of the sampling frequency were applied to both signals to reduce the acquired noise.

The chosen sample rate was progressively increased according to the signal frequency, as it increased as well. For the sake of clarity, Table 4 summarizes the considered test frequencies $\left(f\right)$, the sampling one $\left(f_{s}\right)$, the number of measurements performed $\left(N\right)$, and the supplied voltage $\left(V_{cal}\right)$.

Given the main characteristics of the LPVTs shown in Table 3, it was reasonably decided to perform temperature tests, setting the thermostatic chamber at room temperature (20 °C), 40 °C, and −5 °C, where the last two values correspond to the upper and bottom temperature limits of the device under test. The temperature range is also aligned and includes the Italian yearly average temperature.

It is also worth noting that to perform room-temperature measurements, the thermostatic chamber was set to 20 °C to ensure the temperature stability for the whole duration of the tests.

For the three temperature tests, the measurement settings presented in Table 4 apply as well. What has to be highlighted when testing vs. temperature is the thermal constant of the device under test. Therefore, a preliminary round of test demonstrated that 3–4 h are not sufficient to for the two tested devices to reach thermal stability. On the contrary, longer intervals (8 h or more) guarantee the correct operation of the devices. For this purpose, before each set of testing vs. frequency, both devices were kept at the specific temperature for 12 h.

## 3.2. Impedance Measurement

A second type of test was performed, in addition to the one in Section 3.1, to complete the available information on the capacitive dividers. In particular, the idea was to obtain the primary and secondary capacitances of the dividers. The setup and the test description are provided analogously to what was done for the previous test.

### 3.2.1. Setup

The measurement setup adopted for measuring the CDs’ impedance consists of:

- An Agilent 4284A precision LCR meter. It features a frequency range from 20 Hz to 1 MHz, whereas the impedance measurement range spans from 0.01 mΩ to 99.9999 MΩ. The overall accuracy of the instrument is in the range of 0.1%.
- The two capacitive dividers under test are described in Section 3.1.1 and in Table 3.

### 3.2.2. Test Description

**Figure 4:**
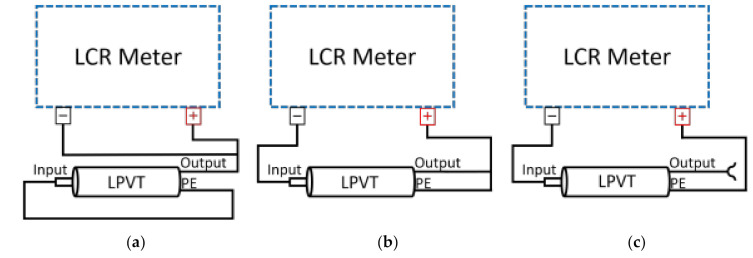
Impedance measurement setup in its three configurations adopted for the tests of the two LPVTs.

The impedance measurements were carried out for both LPVTs at room temperature (20 °C) and at a 10 V voltage supplied by the LCR meter. Three specific test configurations were assessed. They are depicted in Figure 4.

They consist of:

- Short-circuiting the primary terminals (Figure 4a), allowing measuring from the secondary terminals $C_{sa}$ the capacitance obtained from the parallel terminals $C_{1}$ and $C_{2}$.

- Short-circuiting the secondary terminals (Figure 4b), allowing measuring from the primary terminals $C_{sb}$ the capacitance that includes $C_{1}$ (see Figure 1) and potential stray capacitances.

- Open-circuit measurement from the primary terminals (Figure 4c), allowing measuring from $C_{sc}$ the capacitance obtained from the series of $C_{1}$ and $C_{2}$.

The three tests lead to the computation of the primary and secondary capacitances ($C_{1}$ and $C_{2}$), and it was done for various frequencies in the range from 100 Hz to 1 MHz. All the listed tests were performed in a series of 100 measurements and repeated several times on different days to ensure measurement repeatability. Therefore, in the Section 4, the presented figures represent the mean of 100 measurements (of one set of measurements because no discrepancies among the sets have been recorded).

The aim of this set of tests was to collect more information from the devices under test that will be used to better comprehend and comment on the results of the ratio measurements vs. frequency and vs. temperature.

## 4. Results and Discussion

### 4.1. Impedance Test Results

As anticipated in the Section 3.1.1 and Section 3.1.2, this section presents the measurement test results performed using the LCR meter.

First, the capacitance values are given in Table 5 and Table 6 for the dividers A and B, respectively. Both tables report the applied frequency $\left(f\right)$ and the measured series capacitance $\left(C_{s}\right)$ in three different testing conditions: short-circuiting the primary voltage $\left(C_{sa}\right)$, short-circuiting the secondary voltage $\left(C_{sb}\right)$, and keeping the secondary voltage in an open circuit $\left(C_{sc}\right)$.

From the results obtained in Table 5 and Table 6, it is evident that $C_{sb}$, and hence the primary capacitance, is barely affected by the frequency, whereas $C_{sa}$, and hence the secondary one, is strongly dependent on it. As for the potential presence of parasitic capacitances and/or the influence of the setup, both LPVTs A and B were tested with the cable provided by the manufacturer. Only for LPVT B, a 1 m BNC cable was added to extend the connection to the acquisition system. However, (i) the BNC cable does not introduce any reactive components that may affect the impedance measurement (negligible inductive part and capacitance <15 pF/m) and (ii) the input impedance of the oscilloscope (13 pF), in parallel with the LPVTs, is almost negligible compared to the measurement results presented in Table 5 and Table 6.

In terms of impedance, Table 7 and Table 8 summarize the results attained for LPVTs A and B, respectively. The first column reports the applied frequency $\left(f\right)$, followed by the primary $\left(Z_{1}\right)$ and the secondary impedance $\left(Z_{2}\right)$. All the results of Table 5, Table 6, Table 7 and Table 8 are written with a number of digits coherent with the accuracy of the measurement instrument.

The impedance results presented above are used in the following section to better understand the transformation ratio measurement results and the behavior of the two devices under test.

### 4.2. Transformation Ratio and Phase Displacement Test Results

The second part of this section presents the outcomes obtained from the frequency sweeps performed at different temperatures. At each frequency point, illustrated in Table 4, the transformation ratio $\left(K\right)$, computed as the ratio between the rms of the output voltage $\left(V_{s}\right)$ and the rms of the input one $\left(V_{p}\right)$, and the phase displacement between the two considered voltages were calculated.

**Figure 5:**
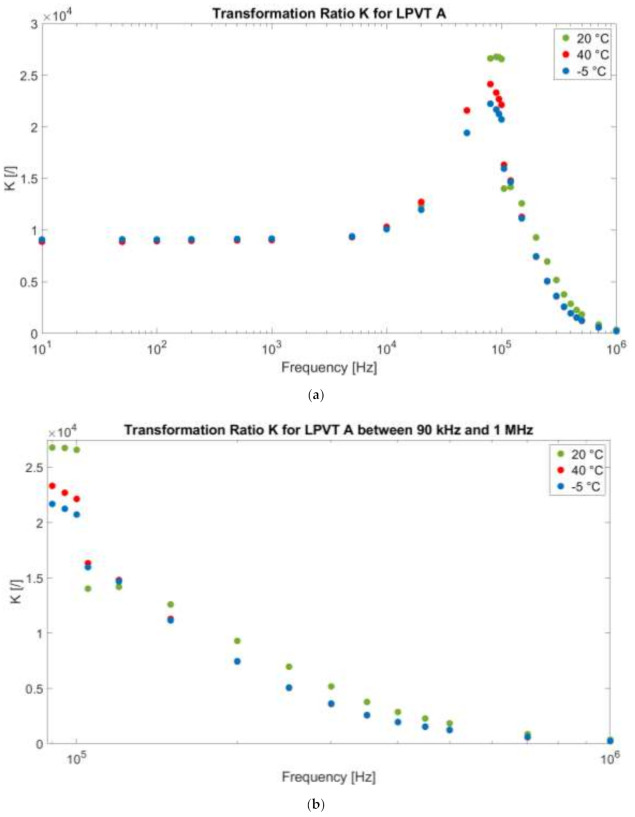
(**a**) Transformation ratio $\left(K\right)$ as a function of the applied frequency f at 20 °C, 40 °C, and −5 °C for LPVT A. (**b**) A zoomed-in portion of the graph from 90 kHz to 1 MHz.

Figure 5a presents the transformation ratio of LPVT A as a function of frequency and temperature (one color for each operating temperature), and Figure 5b is only a zoomed-in portion of the graph to better focus on the high frequencies.

The first comment is dedicated to the 20 °C operation of LPVT A. From the graph, it is clear that K, which is defined at 50 Hz (and it is 9017), remains constant up to 5 kHz, where it starts increasing until it reaches a maximum of 26,790 at 90 kHz. This peak of K is reasonably due to a self-resonance inside of the divider, as better explained in what follows. Afterward, as the frequency increases, K decreases to 353.1 at 1 MHz. In other words, between 0.09 and 1 MHz, the divider loses its scaling capabilities, as shown in Figure 5b.

However, this is not a drawback and could even become a benefit. In other words, considering that PD signals and other disturbances typically have very small amplitudes (a fraction of a percentage compared to the main signal), the fact that the divider does not further scale them is an advantage for their detection and measurement. Therefore, as far as LPVT behavior is known, at all frequencies, one can exploit it for multiple purposes.

Detailing the self-resonance phenomenon, from Table 5 and Table 7, it can be concluded that the self-resonance affects the secondary capacitance of the divider. This is coherent with the conventional way of designing MV capacitive dividers. In fact, the secondary capacitor is a high-capacitance commercial capacitor that, aside from being a very expensive one, is affected by low accuracy and stability. On the contrary, the low-capacitance primary capacitor is obtained from the shell-case of the divider, exploiting its dielectric features. The resins or insulating materials adopted for the MV cases of the divider have minimum requirements of insulation and dielectric properties that ensure minimum standards of accuracy to the primary capacitance.

The second comment is relevant to the temperature. As can be seen from the graph, at rated and low frequencies, the effect of temperature is almost negligible. However, what is really affected by temperature is the self-resonance phenomenon. Consequently, the resonance frequency moves from almost 90 kHz at 20 °C to 80 kHz at both −5 °C and 40 °C temperatures. This behavior can be explained as a variation, and hence a temperature dependency, of the secondary capacitor of the divider. As a result, it can be concluded that the cheaper and the more inaccurate the secondary capacitor is, the higher will be the divider dependency on external influence factors like temperature.

**Figure 6:**
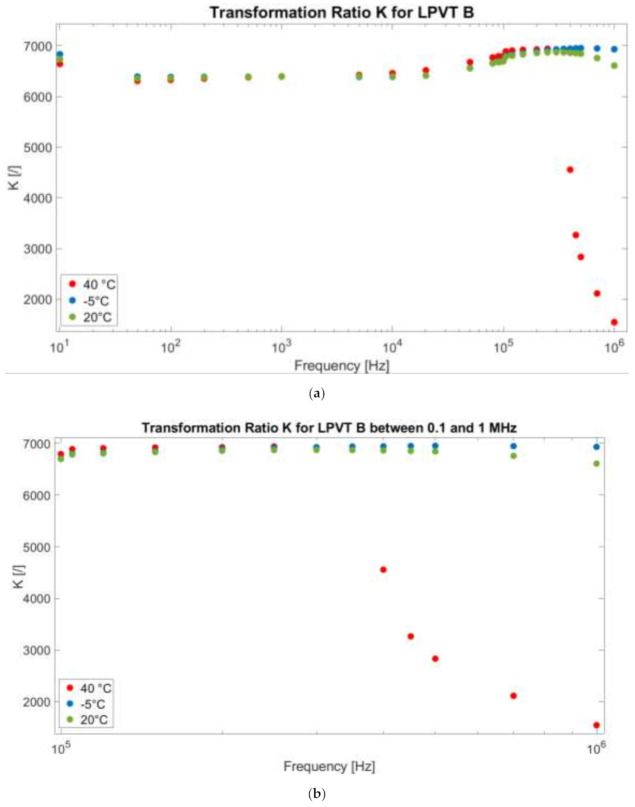
(**a**) Transformation ratio $\left(K\right)$ as a function of the applied frequency f at 20 °C, 40 °C, and −5 °C for LPVT B. (**b**) A zoomed-in portion of the graph from 0.1 to 1 MHz.

Figure 6 presents in a similar way the results related to LPVT B. In the graph, the same color code as in Figure 5 is used.

Note that, at a glance, it is possible to appreciate a completely different behavior compared to LPVT A. First, if the 20 °C case is analyzed, it can be stated that the self-resonance effect is not so evident. Therefore, the scaling behavior of the divider is almost constant in the entire frequency range from 50 Hz to 1 MHz. Consequently, at 20 °C, the high-frequency components measured by the divider are scaled the same way as the power frequency component, resulting in a sort of filtering effect.

Turning to the behavior over temperature, a behavior similar to the 20 °C one is recorded for the operation at −5 °C. In fact, a slight difference can be seen only at a very low frequency (10 Hz) and at the very high ones (around 1 MHz). Therefore, it can be concluded that cold temperatures do not really influence LPVT B, as expected from the rated values listed in Table 3.

What is more interesting is the divider behavior at 40 °C. As can be seen from the graph, the high temperature drastically changes the behavior of the divider at high frequencies. In particular, from 100 kHz, the ratio suffers a slight resonance effect (more visible in the zoomed-in portion of the graph presented in Figure 6b), which results in values K 3 orders of magnitude lower than the one at 50 Hz.

It can be easily concluded that high temperature moves the self-resonance phenomenon on the frequency axis to a lower-frequency value.

**Figure 7:**
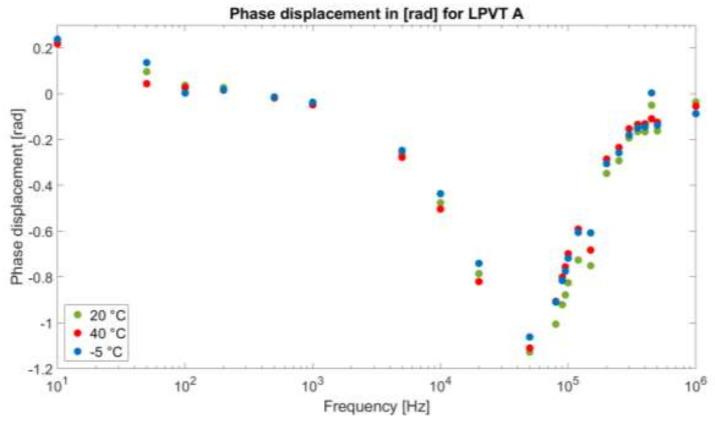
Phase displacement between $\left(V_{s}\right)$ and $\left(V_{p}\right)$ expressed in radians for LPVT A as a function of the frequency f at different temperatures.

**Figure 8:**
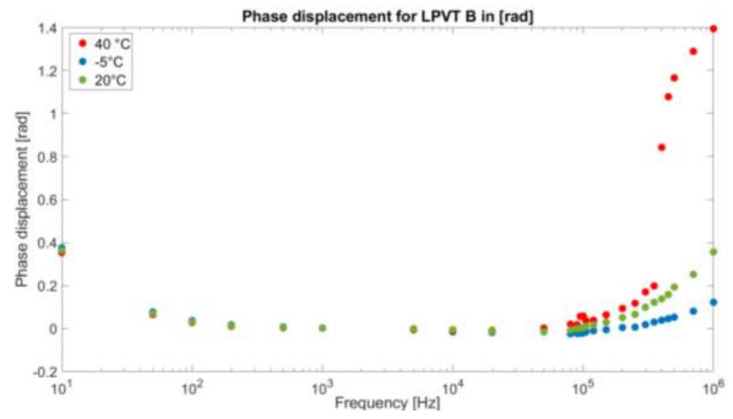
Phase displacement between $\left(V_{s}\right)$ and $\left(V_{p}\right)$ expressed in radians for LPVT B as a function of the frequency f at different temperatures.

As for the phase displacement of the devices under test, Figure 7 and Figure 8 present them, in radians, for A and B, respectively. Compared to the ratio, the phase displacement behaves in a completely different way.

At a glance, the temperature is not an influencing quantity in this case, resulting in a similar behavior of the phase displacement also for −5 °C and 40 °C. Only for LPVT B at 40 °C, the variation measured for the ratio is also present in the phase displacement, confirming that such a value of temperature is critical for the device.

Specifically, Figure 7 presents a behavior that resembles quite well the one of a typical phase displacement in a capacitive divider in which $C_{2}$ is affected by the self-resonance phenomenon. Indeed, the general trend consists of a phase that starts around 0 radians; decreases, reaching a minimum value (theoretically −π) around the resonance frequency; and then increases back to its starting value. This behavior is given in Figure 7.

On the other hand, Figure 8 illustrates a coherent behavior to Figure 7. Nevertheless, the phase displacement changes in a much smoother way with respect to the one of LPVT A.

This can be justified by the fact that the resonance itself of the device under test is much less visible, as shown previously in Figure 6a,b.

Summarizing the above findings, the proposed simple characterization procedure, which provides a sort of modeling of the LPVT’s behavior, allows one to:

- Determine the temperature dependencies of the capacitive dividers, helping one to understand how their behavior, and hence their accuracy, changes vs. temperature.
- Determine the frequency dependency of the capacitive dividers. This result is significant for exploiting the measurement capabilities of the dividers outside their design range. In particular, it has been demonstrated whether it is possible to measure high-frequency components according to the divider behavior.
- Determine the combined effect of temperature and frequency. In fact, the simultaneous presence of the two influencing quantities may or may not change, as presented, the accuracy parameters of the capacitive dividers.

Finally, it can be concluded that with a proper but simple characterization process, SOs and researchers may exploit existing capacitive dividers, designed for operating at specific frequencies, for measuring voltage phenomena at various frequencies (typically fast transients or PD analysis).

## 5. Conclusions

This work aimed to model passive capacitive dividers in a wide range of frequencies and at various temperatures. The motivation of the work came from the high availability of voltage sensors installed along a distribution network. The idea was to exploit them not only for voltage measurement at a power frequency but also for power quality evaluation and high-frequency-disturbance detection. Therefore, this work presented a measurement setup and a set of tests to assess the behavior of two off-the-shelf capacitive dividers. The results, presented in terms of ratio and impedance of the dividers, demonstrated that when properly studied and characterized, a capacitive divider can be used for measuring voltages in a wide band of frequencies and temperatures. Furthermore, the not-negligible effect of temperature on the devices highlighted the need of appropriate models, to be used by system operators, to avoid any wrong measurements or accuracy degradation. As a final note, this characterization procedure may (i) be adopted by manufacturers to be included in the device datasheet to provide additional information about its use and (ii) be included in the standards as a simple test to highlight the features of an LPIT.

## Figures and Tables

**Table 1:** Accuracy specifications of the Datron Wavetek 4800 calibrator for a 10 V range.

Frequency Band (Hz)	Accuracy (ppm + mV)
10–31	135 + 300
32–330	80 + 200
300–33 k	80 + 100
30 k–100 k	150 + 200
100 k–330 k	400 + 1
300 k–1 M	0.26% + 5

**Table 2:** Accuracy specifications of the Datron Wavetek 4800 calibrator for the 100 V range.

Frequency Band (Hz)	Accuracy (ppm + mV)
10–31	190 + 3
32–330	120 + 2
300–10 k	80 + 1
10 k–33 k	90 + 2
30 k–100 k	300 + 3
100 k–330 k	860 + 50
300 k–1 M	0.95% + 130

**Table 3:** Main characteristics of the two passive capacitive low-power voltage transformers (LPVTs) under test.

LPVT	$V_{pr}(V)$	$V_{sr}(V)$	AC (/)	$f_{r}(\mathrm{Hz})$	$T_{R}({}^{\circ}C)$
A	20,000/$\sqrt{3}$	2/$\sqrt{3}$	0.5	50/60	−5 to + 40
B	20,000/$\sqrt{3}$	3.25/$\sqrt{3}$	0.5	50/60	−40 to + 80

**Table 4:** Main settings of the frequency sweep test.

*f* (Hz)	$f_{s}(\mathrm{MSa}/s)$	*N* (/)	$V_{cal}\;(V)$
10	1.25	200	200
50	1.25	200	200
100	1.25	200	200
200	1.25	200	200
500	1.25	400	200
1000	1.25	400	200
5000	1.25	400	200
10,000	1.25	400	200
20,000	1.25	400	200
50,000	5	400	200
80,000	5	400	200
90,000	5	400	200
95,000	5	400	200
100,000	12.5	400	200
105,000	12.5	400	20
120,000	12.5	400	20
150,000	12.5	400	20
200,000	12.5	400	20
250,000	12.5	400	20
300,000	12.5	400	20
350,000	25	400	20
400,000	25	400	20
450,000	25	400	20
500,000	25	400	20
700,000	125	400	20
1,000,000	125	400	20

**Table 5:** Series capacitance test results for LPVT A at 10 V.

f (Hz)	$C_{sa}\;(\mathrm{pF})$	$C_{sb}\;(\mathrm{pF})$	$C_{sc}\;(\mathrm{pF})$
100	106.6 $\times 10^{3}$	36.80	35.8
500	93.6 $\times 10^{3}$	36.74	35.55
1000	67.8 $\times 10^{3}$	36.64	35.4
10,000	2.15 $\times 10^{3}$	36.31	35.1
50,000	425.1	36.07	34.9
100,000	370.1	35.97	34.7
300,000	354.6	35.79	34.57
1,000,000	364.6	35.58	34.37

**Table 6:** Series capacitance test results for LPVT B at 10 V.

*f* (Hz)	$C_{sa}\;(\mathrm{nF})$	$C_{sb}\;(\mathrm{pF})$	$C_{sc}\;(\mathrm{pF})$
100	39.72	10.65	8.28
500	39.63	10.57	8.23
1000	39.58	10.56	8.16
10,000	39.35	10.43	8.08
50,000	38.46	10.32	8.07
100,000	36.3	10.29	8.06
300,000	21.13	10.27	7.96
1,000,000	4.607	10.19	7.91

**Table 7:** Modules of the obtained primary $\left(Z_{1}\right)$ and secondary $\left(Z_{2}\right)$ impedances for LPVT A at 10 V at room temperature.

f (Hz)	$Z_{1}(Ω)$	$Z_{2}(Ω)$
100	4.325 $\times 10^{7}$	1.494$\;\times 10^{4}$
500	8.664$\;\times 10^{6}$	3.402$\;\times 10^{3}$
1000	4.344$\;\times 10^{6}$	2.349$\;\times 10^{3}$
10,000	4.383$\;\times 10^{5}$	7.530$\;\times 10^{3}$
50,000	8.825$\;\times 10^{4}$	8.182$\;\times 10^{3}$
100,000	4.425$\;\times 10^{4}$	4.763$\;\times 10^{3}$
300,000	1.482$\;\times 10^{4}$	1.664$\;\times 10^{3}$
1,000,000	4.473$\;\times 10^{3}$	4.837$\;\times 10^{2}$

**Table 8:** Modules of the obtained primary $\left(Z_{1}\right)$ and secondary $\left(Z_{2}\right)$ impedances for LPVT B at 10 V at room temperature.

f (Hz)	$Z_{1}(Ω)$	$Z_{2}(Ω)$
100	1.494 $\times 10^{8}$	4.008 $\times 10^{4}$
500	3.011$\;\times 10^{7}$	8.034 $\times 10^{3}$
1000	1.507$\;\times 10^{7}$	4.022 $\times 10^{3}$
10,000	1.526$\;\times 10^{6}$	4.046 $\times 10^{2}$
50,000	3.084$\;\times 10^{5}$	82.79
100,000	1.547$\;\times 10^{5}$	43.86
300,000	5.166$\;\times 10^{4}$	25.12
1,000,000	1.562$\;\times 10^{4}$	34.62

## Data Availability

Not applicable.
